# The Combinatorial Effect of Cisplatin and Moxibustion on Tumor Growth Inhibition with Special Reference to Modulation of the Immune Microenvironment in Lewis Lung Cancer Mice

**DOI:** 10.1155/2020/3170803

**Published:** 2020-12-29

**Authors:** Bin Wang, Jin Huang, Shanshan Li, Zhanyu Pan, Yongming Guo, Yinli Yang, Ling Li, Cong Wang, Yinan Gong, Jiaqi Wang, Shanshan Lu, Zhifang Xu, Yi Guo

**Affiliations:** ^1^Tianjin Medical University Cancer Institute and Hospital, National Clinical Research Center for Cancer, Key Laboratory of Cancer Prevention and Therapy, Tianjin's Clinical Research Center for Cancer, Tianjin 300060, China; ^2^Research Center of Experimental Acupuncture Science, Tianjin University of Traditional Chinese Medicine, Tianjin 301617, China; ^3^School of Acupuncture & Moxibustion and Tuina, Tianjin University of Traditional Chinese Medicine, Tianjin 301617, China; ^4^School of Traditional Chinese Medicine, Tianjin University of Traditional Chinese Medicine, Tianjin 301617, China

## Abstract

**Objective:**

As a first-line treatment for non-small cell lung cancer (NSCLC), the efficacy of chemotherapy is still unsatisfactory. Moxibustion has been shown to improve the side effects of radiotherapy and chemotherapy and regulate immune function. This study aimed to explore the antitumor effects and potential mechanisms of combinatorial cisplatin and moxibustion treatment for NSCLC by targeting the tumor microenvironment.

**Methods:**

Lewis lung cancer (LLC)-bearing mice were induced and treated with cisplatin or/and moxibustion at ST36 (Zusanli), and the growth, weight, and area of the tumor were evaluated. The numbers of various T cell subsets and myeloid cells in the tumor were assessed by flow cytometry, and the gene expression of related markers and cytokines was detected with real-time quantitative polymerase chain reaction (RT-qPCR). In addition, the tumor vascular structure was investigated using CD31 and *α*-smooth muscle actin (*α*-SMA) immunofluorescence staining. The expression of the vascular endothelial growth factor (VEGF) and hypoxia-inducible factor-1*α* (HIF-1*α*) was detected by immunohistochemical staining.

**Results:**

Both cisplatin and moxibustion inhibited LLC tumor growth and reduced both the tumor area and weight, with the combinatorial therapy showing superior outcomes. Moxibustion upregulated the infiltration of CD4^+^ T cells and Th1 cells in the tumor, and the combinatorial therapy increased the proportion of CD8^+^ cytotoxic T cells (CTLs), CD4^+^T cells, Th1, Th9 cells, and M1 macrophages, as well as the expression of *Cd69*, *Ifng*, and *Cd86* mRNA. The combinatorial therapy improved vascular normalization by increasing both the microvessel density (MVD) and pericyte coverage (*α*-SMA area density) and inhibiting the expression of the VEGF.

**Conclusions:**

Combinatorial cisplatin and moxibustion treatment inhibited the LLC tumor growth by mechanisms related to the improvement of the tumor immune microenvironment and vascular normalization, providing an effective combinatorial therapy beneficial for patients with NSCLC.

## 1. Introduction

Lung cancer is one of the most common and aggressive tumors worldwide with only a 15% five-year survival rate. Non-small cell lung cancer (NSCLC) accounts for between 80% and 85% of all lung cancers [1, 2]. More than 80% of newly diagnosed lung cancer patients receive chemotherapy, alone, or combined with radiation [3]. However, the treatment is often unsatisfactory, with the objective remission rate of platinum-containing dual-drug chemotherapy being less than 25%. Possible reasons include resistance to chemotherapeutic drugs [4], as well as the induction of myelosuppression and immunosuppression by high-dose chemotherapeutic drugs, contributing to tumor progression and metastasis [5]. Thereby, the effects of chemotherapy as a first-line cancer treatment still require investigation.

The tumor microenvironment is complex and includes blood vessels and immune and other cells, as well as factors influencing the proliferation and apoptosis of the tumor cells [6]. Tumor cells can trigger a variety of immunosuppressive cascade reactions, including inducing immature immune cells to differentiate into immunosuppressive phenotypic cells [7]. At the same time, vascular abnormalities and dysfunction within a solid tumor can lead to low oxygen, glycolysis, low pH, high blood vessel permeability, and interstitial pressure [8], which impair the infiltration of antitumor immune effector cells and chemotherapy drugs to the tumor site [9]. Therefore, alleviating the immunosuppressive and vascular abnormalities may be strategies to improve chemotherapy for cancer patients.

Moxibustion is a popular traditional Chinese medicine, consisting of a drug extracted from the mugwort herb, which treats disease through direct or indirect thermal stimulation of a particular acupoint [10, 11]. Moxibustion has been demonstrated to stimulate both innate and adaptive immunity [12]. Specifically, several clinical studies have reported that moxibustion can improve both the immune function and quality of life of patients with different cancers, including lung cancer and cervical cancer [11, 13–15]. However, the mechanism of targeting the tumor microenvironment remains unknown.

This study investigated the inhibition of tumor growth produced by a chemotherapeutic drug (cisplatin) and/or moxibustion, elucidating the mechanism involved in terms of the tumor immune microenvironment and vascular system, to improve the treatment of lung cancer.

## 2. Materials and Methods

### 2.1. Mice

C57/BL6 male mice (6 weeks old, body weight 18–24 g, *n* = 80) were purchased from Beijing Wei Tong Li Hua Laboratory Animal Technology Co., Ltd., Beijing, China. All mice were maintained on a 12 h light/dark cycle, with constant temperature (24°C) and humidity (40–50%) conditions, and with free access to food and water. All manipulations and procedures were carried out in accordance with the Guidance Suggestions for the Care and Use of Laboratory Animals produced by the Ministry of Science and Technology of China. The experiments were approved by the Animal Care and Use Committee of Tianjin University of Traditional Chinese Medicine (Permit Number: TCM-LAEC2019057).

### 2.2. Cell Culture

Lewis lung cancer (LLC) cells were purchased from Shanghai Cell Biology Institute (Shanghai, China) and were cultured with Dulbecco's Modified Eagle Medium (Gibco, Waltham, MA, USA) supplemented with 10% fetal bovine serum (FBS), 100 *μ*g/mL penicillin, and 0.1 mg/mL streptomycin and were maintained in a humidified chamber at 37°C in a 5% CO_2_ atmosphere in Tianjin Cancer Institute.

### 2.3. Determination of Tumor Growth

**Figure 1:**
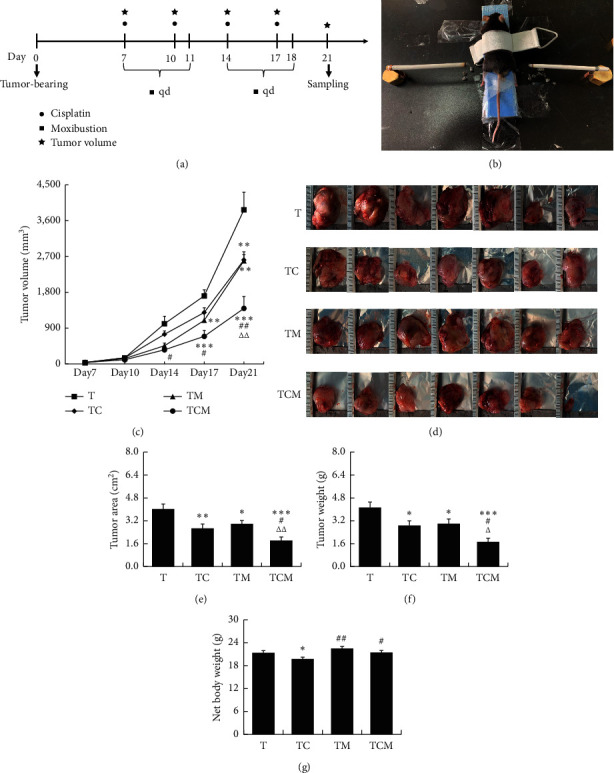
The effect of combinatorial cisplatin and moxibustion treatment on tumor growth in LLC-bearing mice. (a) Flowchart of experimental interventions. ^●^Cisplatin, ^▪^moxibustion, and ^★^tumor volume test. LLC cells were transplanted into mice on day 0. The mice in the cisplatin (TC) and the combinatorial therapy groups (TCM) were treated with cisplatin on days 7, 10, 14, and 17. Mice in the moxibustion (TM) and the combination groups (TCM) were treated with moxibustion on days 7‐11 (once a day). The tumor volume was measured on days 7, 10, 14, 17, and 21, and mice were sacrificed on day 21 for sample collection. (b) Illustration of moxibustion intervention. (c) Tumor growth curve analysis of the LLC-bearing mice in each group at the corresponding time points (days 7, 10, 14, 17, and 21; *n* = 7). (d) Images of the dissected tumors from each group (*n* = 7). (e) The area of tumor tissues in each group (*n* = 7). (f) Tumor weight after removal from mice at day 21 (*n* = 7). (g) Effects of cisplatin and moxibustion on the net body weight of LLC-bearing mice. Data in (c), (e), (f), and (g) are mean ± SEM, ^*∗*^*P* < 0.05, ^*∗∗*^*P* < 0.01, and ^*∗∗∗*^*P* < 0.001*vs*. T; ^#^*P* < 0.05 and ^##^*P* < 0.01*vs*. TC; ^Δ^*P* < 0.05 and ^△△^*P* < 0.01*vs*. TM.

After the mice had been acclimated for one week, 1 × 10^5^ LLC cells resuspended in 0.1 mL phosphate-buffered saline (PBS) were subcutaneously implanted into the right groin [16]. Tumor dimensions were measured by digital calipers at days 7, 10, 14, 17, and 21 (Figure 1(a)), and the tumor volume (mm^3^) was calculated as follows: (length × width^2^)/2 [17]. At day 21, LLC-bearing mice were weighed and deeply anesthetized with 4% isoflurane with oxygen as carrier (Shenzhen RWD Life Technology Co., Ltd. China) before sacrifice. The intact tumor tissue was removed and weighed, and the net body weight was calculated (net body weight = body weight−tumor tissue weight) and photographed with a ruler as a reference. The tumor area was calculated with Adobe Photoshop CC 2015 using the formula, tumor actual area = tumor pixel × actual area of the whole image/pixel of the whole image.

### 2.4. Cisplatin and Moxibustion Intervention

After one week, the tumor-bearing mice were randomly divided into the tumor group (T), cisplatin group (TC), moxibustion group (TM), and combined cisplatin and moxibustion group (TCM, *n* = 7/group). Cisplatin (Jiangsu Haosen Pharmaceutical Group Co., Ltd., China) was administered at 3 mg/kg in 0.9% sodium chloride solution by intraperitoneal injection on days 7, 10, 14, and 17 in mice in the TC and TCM groups, while mice in the T and TM groups were intraperitoneally injected with a 0.9% sodium chloride solution (Figure 1(a)).

Moxibustion was carried out for 15 min, five times per week for two weeks (Figure 1(a)) [18]. Before moxibustion, the fur on the moxibustion region was shaved to expose the bilateral ST36 (Zusanli acupoint, located 2 mm lateral to the anterior tubercle of the tibia in the anterior tibial muscle and 4 mm distal to the knee joint lower point). The mice were immobilized with a homemade modified moxibustion fixer (Mouse fixator, Chengdu University of Chinese Medicine, Patent No. ZL201220746188). During the moxibustion process, the distance between the skin of ST36 and the lighted end of the moxa sticks (length: 120 mm, diameter: 5 mm, Nanyang Hanyi Moxibustion Technology Development Co., Ltd., China) was controlled within 1–1.5 cm. At the same time, the distance was adjusted in time by an electronic temperature meter to control the temperature in the 42 ± 2°C range. All procedures were performed at room temperature around 24°C (Figure 1(b)). Mice in the T and TC groups without the moxibustion operation were placed in the moxibustion fixer under the same conditions.

### 2.5. Flow Cytometry Assay (FCM)

The tumor tissues were cut into pieces and digested in RPMI-1640 medium with collagenase IV (2 mg/ml, Sigma, Deisenhofen, Germany) and hyaluronidase (0.2 mg/mL, Sigma, Deisenhofen, Germany) in the shaker at 200 rpm/min for 1 h at 37°C, following which the suspension was filtered through a 70 *μ*m mesh cell sieve. A lysing buffer (BD Bioscience, Franklin Lakes, New Jersey, USA) was used to remove red blood cells. The cell resuspension was divided into three panels. In panel 1 of regulatory T cells (Tregs) staining setting, single cells were incubated with cell membrane markers including CD3-FITC, CD4-PE-Cy5, CD25-APC-Cy7, and CD45-PE (Biolegend, San Diego, California, USA) for 20 min in the dark at room temperature and then fixed in fixation/permeabilization working solution overnight. After washing with the permeabilization buffer, Foxp3-eFluor 450 (Biolegend) was added for intracellular staining. Samples were then washed again before resuspension in 0.5 mL PBS containing 1% FBS. Panel 2 consisted of CD8^+^ cytotoxic T cells (CTLs) and CD4^+^ T cell subsets and panel 3 of myeloid cells. Single-cell resuspensions were stained with CD3-FITC, CD4-PE-Cy5, CD8-Brilliant Violet 421™, CD45-APC-Cy7, CCR6-Brilliant Violet 605™, CXCR3-PE, and CCR4-PE-Cy7 (Biolegend) and CCR10-APC (R&D Systems, Minneapolis, USA) for panel 2 and CD45-PE, CD11b-FITC, CD86-Brilliant Violet 421™, CD206-APC, CD11c-PerCP/Cy5.5, and Gr-1-PE-cy7 (Biolegend, San Diego, California, USA) for panel 3. Cells were stained for 20 min in the dark at room temperature. Samples were then washed twice before resuspension in 0.5 mL PBS containing 1% FBS. Acquisition and data analysis were conducted on an Attune NxT Flow Cytometer (Thermo Fisher Scientific, Waltham, MA, USA). The data were analyzed as follows: positive cell events (%) = (the events in target gate/the total single cell) × 100.

### 2.6. Real-Time Quantitative Polymerase Chain Reaction (RT-qPCR)

Tumor tissues (*n* = 6) were frozen in liquid nitrogen (−195.79°C) and stored at −80°C. The total RNA was extracted by the TRIzol reagent (Thermo Fisher Scientific China, Shanghai, China) in accordance with the manufacturer's protocol [19]. The RNA concentration was measured by the Agilent Bioanalyzer 2100 (Agilent Technologies, Santa Clara, CA, US), and the total RNA was used for reverse transcription by the PrimeScript RT Master Mix Perfect Real Time (Takara Bio, Inc., Otsu, Japan) following the manufacturer's protocol. The cDNA synthesis was amplified by the SYBR™ Select Master Mix (Applied Biosystem, Thermo Fisher Scientific, Inc.) for RT-qPCR according to the manufacturer's protocol. Applied ABI QuantStudio 3 Real-Time PCR System (Applied Biosystems; Thermo Fisher Scientific Inc.) was used for RT-qPCR under the following conditions: 95°C for 30 sec, followed by 40 cycles of 95°C for 5 sec and 60°C for 30 sec, and finally, the melt curve stage (95°C for 15 sec, 60°C for 1 min, and 95°C for 15 sec). The associated primers were synthesized by Shanghai Sangon Biotech Co. Ltd. (Shanghai, China) and are listed in Table 1. *Actb* was used as an endogenous reference. The relative gene expression was calculated by the double-standard curve method.

### 2.7. Immunohistochemical (IHC) Staining

The tumor tissues were fixed with 4% paraformaldehyde (PFA, pH 7.4) and embedded in paraffin. The embedded tissue was cut into 4 *μ*m sections and deparaffinized. After antigen retrieval with citric acid antigen repair buffer (pH 6.0) using the heating method, the slides were incubated in 3% hydrogen peroxide solution and blocked with 3% bovine serum albumin (BSA) (Servicebio, Wuhan, China). Sections were incubated with either the rabbit antivascular endothelial growth factor (VEGF) or rabbit antihypoxia inducible factor-1*α* (HIF-1*α*) (Servicebio) at 4°C overnight, followed by incubation with HRP-labeled anti-rabbit IgG (Servicebio) as the secondary antibody for 60 min. Sections were incubated with DAB until the brown positive signal was observed. After staining the nucleus with hematoxylin, the sections were dehydrated and sealed, followed by imaging with a normal fluorescence microscope (NIKON Eclipse Ci, Japan) and imaging system (NIKON digital sight DS-FI2, Japan).

### 2.8. Immunofluorescence (IF) Staining

Tissues were fixed and sectioned as described above Section 2.7. After antigen retrieval in EDTA antigen repair buffer (pH 8.0) with heating, the sections were blocked in 3% BSA (Servicebio) and incubated with the primary antibodies rabbit anti-platelet and endothelial cell adhesion molecule-1 (CD31) (Abcam, Cambridge, UK) and mouse anti-*α*-smooth muscle actin (*α*-SMA) (Servicebio) overnight at 4°C. After washing, the sections were incubated with CY3-labeled anti-rabbit IgG (Servicebio) or FITC-labeled anti-mouse IgG (Servicebio) as secondary antibodies for 60 min. Nuclei were stained with 4′, 6-diamidino-2-phenylindole (DAPI). The sections were sealed with antifluorescence quenching sealing solution and observed and photographed under a fluorescence microscope (NIKON Eclipse Ci, Japan) and imaging system CaseViewer 2.0 (Panoramic 250/MIDI, 3D HISTECH, Hungary).

### 2.9. Histological Analysis

The immunohistochemical and immunofluorescence results were analyzed by Image-Pro Plus 6.0 software (Media Cybernetics, Inc., Rockville, MD, USA). The section within each group (*n* = 3) of randomly selected perspective in 3 pictures. For area density semiquantitative analysis, the accumulation of positive area integrated optical density (IOD) and the tissue total pixel area of each photograph were analyzed, and area density (%) = (IOD/pixel area) × 100. The semiquantitative scoring of the VEGF and HIF-1*α* was carried out as follows: parameter *A* was decided by the degree of staining intensity with 0 representing no color, consistent with the background color; 1 was light yellow, slightly higher than the background color; 2 was brown, which was significantly higher than the background color; and 3 was brown. Parameter *B* of the percentage of positive cells was scored as follows: 0 as negative, 1 as less than 10%, 2 as 11%–50%, 3 as 51%–75%, and 4 as more than 75%. The semiquantitative score was represented as *A* × *B*. The microvessel density (MVD) was calculated as the mean number of CD31^+^-stained vessels in three images from each group. Vessels were independent blood vessels that could be separated from the adjacent vessels, tumor cells, and stromal components. CD31^+^-stained sections were scanned at 200 magnification to determine the most vascularized area (hot spot) of the tumor, and the MVD was calculated as the mean number of hot spots examined in three fields [20].

### 2.10. Statistical Analysis

All data are expressed as means ± SEM and analyzed by analysis of variance (ANOVA) for independent samples comparing the differences between groups. If the data were normally distributed, the least significant difference (LSD) method was used if in agreement with the homogeneity test of variance, while Dunnett's T3 method was used if not in agreement with the homogeneity test of variance. It the data did not conform to the normal test, nonparametric tests using Kruskal–Wallis were applied with SPSS (Version 23.0, IBM Corp., Armonk, NY, USA). Data from the immune cell population in FCM analysis showed a skewed distribution and were multiplied by 100 converted base-10 logarithms to achieve a normal distribution for statistical analysis. *P* < 0.05 was considered statistically significant.

## 3. Results

### 3.1. Combinatorial Cisplatin and Moxibustion Inhibited Tumor Growth in LLC-Bearing Mice

To explore whether moxibustion could enhance the antitumor effect of cisplatin, we assessed the tumor growth curve dynamically in LLC-bearing mice treated with cisplatin, moxibustion, and combinatorial therapy. As shown in Figure 1(c), the mean tumor size was 33.76 ± 4.76 mm^2^, and there was no difference in the tumor volume between the groups before the intervention (one week after tumor cell transplantation). By day 14 (one week after the intervention and two weeks after the tumor cell transplantation), combinatorial therapy displayed an enhanced antitumor effect over cisplatin (^#^*P* < 0.05* vs*. TC). By day 17, moxibustion intervention had reduced the tumor size (^*∗∗*^*P* < 0.01* vs*. T) with the combinatorial therapy inhibiting tumor growth to a greater extent (^*∗∗∗*^*P* < 0.001* vs*. T and ^#^*P* < 0.05* vs*. TC). By day 21, the tumor volume was decreased to greater extents in both the cisplatin or moxibustion treated mice than that in the tumor group (^*∗∗*^*P* < 0.01* vs*. T), and the tumor volume was further inhibited by combinatorial therapy (^*∗∗∗*^*P* < 0.001* vs*. T, ^##^*P* < 0.01* vs*. TC, and ^△△^*P* < 0.01* vs*. TM). A similar pattern was observed in the images of tumors isolated from mice at day 21, as shown in Figure 1(d). Moreover, measurement of the tumor area (^*∗∗*^*P* < 0.01 TC *vs*. T and ^*∗*^*P* < 0.05 TM *vs*. T, Figure 1(e)) and weight (^*∗*^*P* < 0.05* vs*. T, Figure 1(f)) demonstrated that tumor growth was significantly inhibited by both cisplatin and moxibustion intervention, and was further decreased by the combinatorial therapy (^*∗∗∗*^*P* < 0.001* vs*. T, ^#^*P* < 0.05* vs*. TC, and ^△△^*P* < 0.01* vs*. TM, Figure 1(e)) and tumor weight (^*∗∗∗*^*P* < 0.001* vs*. T, ^#^*P* < 0.05* vs*. TC, and ^△^*P* < 0.05* vs*. TM, Figure 1(f)). The net body weight of the tumor-bearing mice were further analyzed, and it was found that both moxibustion and the combinatorial therapy improved the condition of the tumor-bearing mice (^*∗*^*P* < 0.05 TC *vs*. T, ^##^*P* < 0.01 TM *vs*. TC, and ^#^*P* < 0.05 TCM *vs*. TC, Figure 1(g)).

### 3.2. Combinatorial Cisplatin and Moxibustion Treatment Regulated T Cell Subsets and Myeloid Cells in the Tumor Immune Microenvironment

**Figure 2:**
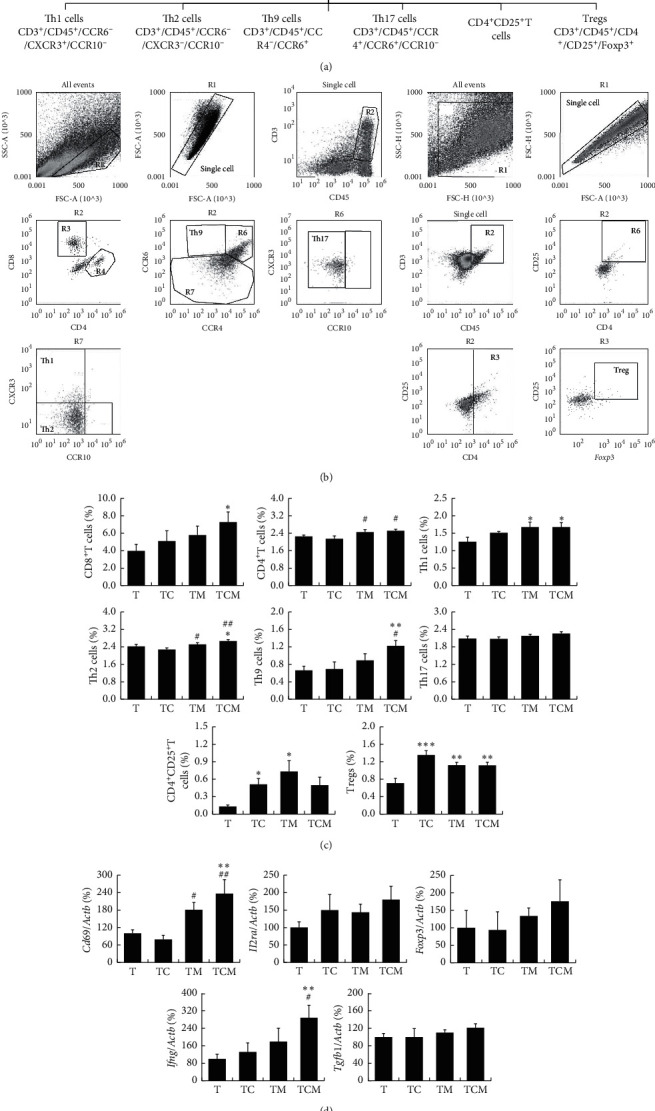
The effect of combinatorial cisplatin and moxibustion treatment on T lymphocyte subsets in the tumor immune microenvironment. (a) Hierarchy and related markers of T cells. (b) Representative FCM patterns used in the present experiments. T cell subpopulations including CD8^+^ CTLs, CD4^+^ T Th1, Th2, Th9, Th17, CD4^+^CD25^+^T cells, and Tregs (R3-CD8^+^ CTLs and R4-CD4^+^ T cells) detected in the current study. (c) Quantification of T cell subset proportions in the tumor site of LLC-bearing mice in indicated treatment groups (*n* = 7). (d) The expression of T cell-related genes including *Cd69, Il2ra, Foxp3, Ifng,* and *Tgfb1* (*n* = 6). Data are mean ± SEM. With the exception of CD8^+^ CTLs, the data of other cell subsets are represented by the log value used as statistics. ^*∗*^*P* < 0.05, ^*∗∗*^*P* < 0.01, and ^*∗∗∗*^*P* < 0.001*vs*. T; ^#^*P* < 0.05 and ^##^*P* < 0.01*vs*. TC.

In the tumor immune microenvironment, infiltrating T lymphocytes are the main cells of the antitumor immune response. They are stimulated by various cytokines to differentiate into CD4^+^ Th cells, CD8^+^ CTLs, and Tregs [21]. As shown in Figures 2(a) and 2(b), several T cell subpopulations were detected by FCM with membrane cell markers (CD3, CD4, CD8, CD25, CD45, CCR4, CCR6, CCR10, and CXCR3) and the nuclear marker *Foxp3*. As shown in Figure 2(c), the proportion of CD8^+^ CTLs (CD3^+^CD45^+^CD8^+^) in the combinatorial group was increased compared with that in untreated tumor-bearing mice (^*∗*^*P* < 0.05* vs*. T), and the proportion of CD4^+^ T cells (CD3^+^CD45^+^CD4^+^) was increased by both moxibustion and combinatorial therapy compared to cisplatin alone (^#^*P* < 0.05* vs*. TC). In addition, increased populations of Th1 cells (CD3^+^CD45^+^CCR6^−^CXCR3^+^CCR10^−^) in the TM (^*∗*^*P* < 0.05* vs*. T) and TCM groups (^*∗*^*P* < 0.05* vs*. T) were observed when compared to those in the *T* group. The percentage of Th2 cells (CD3^+^CD45^+^CCR6^−^CXCR3^−^CCR10^−^) was higher in the TM (^#^*P* < 0.05* vs*. TC) and combinatorial therapy groups (^*∗*^*P* < 0.05* vs*. T and ^##^*P* < 0.01* vs*. TC) than that in the cisplatin-treated and/or untreated tumor-bearing mice. Moreover, the proportion of Th9 cells (CD3^+^CD45^+^CCR4^−^CCR6^+^) was increased in the combinatorial group (^*∗∗*^*P* < 0.01* vs*. T and ^#^*P* < 0.05* vs*. TC). There were no differences in Th17 cells (CD3^+^CD45^+^CCR4^+^ CCR6^+^CCR10^−^) between the groups. In addition, CD4^+^CD25^+^T cells were elevated in the cisplatin and moxibustion groups (^*∗*^*P* < 0.05* vs*. T). Furthermore, there was an increase in the Tregs subset (CD3^+^CD45^+^CD4^+^CD25^+^Foxp3^+^) in the cisplatin (^*∗∗∗*^*P* < 0.001* vs*. T), moxibustion (^*∗∗*^*P* < 0.01* vs*. T), and combinatorial therapy (^*∗∗*^*P* < 0.01* vs*. T) groups compared with that in the untreated tumor-bearing mice. When the expression of genes associated with T cells in the tumor microenvironment in each group was examined, moxibustion and combinatorial treatment increased the expression of *Cd69* (^*∗∗*^*P* < 0.01 TCM *vs*. T, ^#^*P* < 0.05 TM *vs*. TC, and ^##^*P* < 0.01 TCM *vs*. TC), a surface marker for activated T cells. The expression of the Treg cell surface markers *Il2ra (Cd25)* and *Foxp3* mRNA showed no difference after moxibustion and chemotherapy intervention. Furthermore, the expression of interferon-gamma (IFN-*γ*), the principal cytokine secreted by Th1 cells, and *Ifng* mRNA was promoted by the combination therapy (^*∗∗*^*P* < 0.01 TCM *vs*. T and ^#^*P* < 0.01 TCM *vs*. TC). However, moxibustion did not affect the expression of *Tgfb1*, which is secreted by immunosuppressive cells such as Th2, Treg cells, and M2 macrophages (Figure 2(d)).

**Figure 3:**
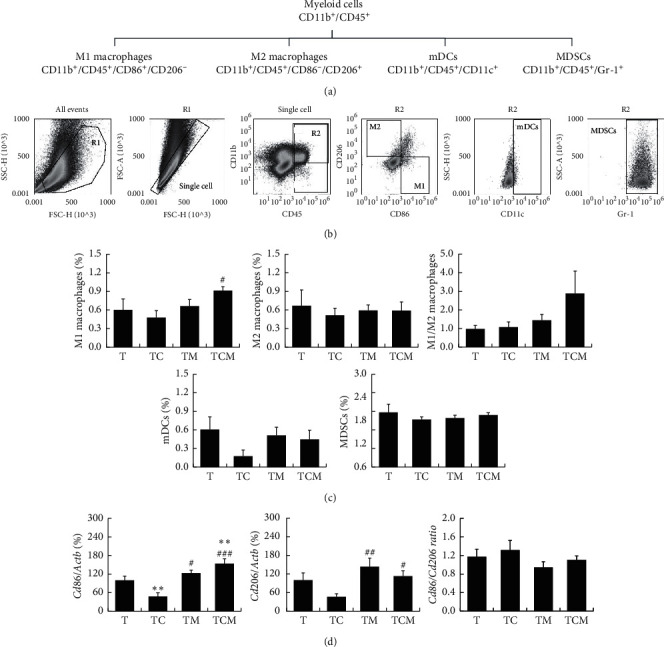
The effect of combinatorial cisplatin and moxibustion treatment on myeloid cell subsets in the tumor immune microenvironment. (a) Hierarchy and related markers of myeloid cell subsets. (b) Representative FCM patterns used in the experiments. Myeloid cell subpopulations included M1 macrophages, M2 macrophages, mDCs, and MDSCs. (c) Quantification of myeloid cell subset proportions in the tumor site of LLC-bearing mice in indicated treatment groups (*n* = 7). (d) The gene expression of macrophage-related markers including *Cd86, Cd206*, and the *Cd86/Cd206* ratio (*n* = 6). Data are mean ± SEM by the log value used as statistics. ^*∗*^*P* < 0.05 and ^*∗∗*^*P* < 0.01*vs*. T; ^#^*P* < 0.05, ^##^*P* < 0.01, and ^###^*P* < 0.001*vs*. TC.

Tumor-infiltrating innate immune cells mainly include natural killer cells, tumor-associated macrophages (TAMs), myeloid dendritic cells (mDCs), and myeloid-derived suppressor cells (MDSCs), most of which can connect innate immunity and adaptive immunity to fight tumor cells [22]. The myeloid cell subsets, including M1 macrophages, M2 macrophages, mDCs, and MDSCs in the tumor, were detected by FCM using a mixture of related membrane cell markers (CD11b, CD45, *CD86, CD206,* CD11c, and Gr-1) as shown in Figures 3(a) and 3(b). Interestingly, the proportion of M1 macrophages (CD11b^+^CD45^+^CD86^+^CD206^−^) was increased with the combinatorial therapy compared with cisplatin treatment (^#^*P* < 0.05* vs*. TC), while there was no difference in M2 macrophages (CD11b^+^CD45^+^CD86^−^CD206^+^) between the groups. The combinatorial therapy treated mice displayed increased tendency of M1/M2 ratio compared with either the moxibustion or cisplatin group. There was no difference in the mDCs (CD11b^+^CD45^+^CD11c^+^) and MDSCs (CD11b^+^CD45^+^Gr-1^+^) proportions between the groups **(**Figure 3(c)). Furthermore, when the gene expressions of the M1 macrophage-related marker *Cd86* and the M2 macrophage-related marker *Cd206* were detected, the expression of *Cd86* was observed to decrease after chemotherapy (^*∗∗*^*P* < 0.01 TC *vs*. T), while both moxibustion therapy and the combinatorial therapy could promote its expression (^*∗∗*^*P* < 0.01 TCM *vs*. T, ^#^*P* < 0.05 TM *vs*. TC, and ^###^*P* < 0.001 TCM *vs*. TC). Whereas moxibustion treatment increased *Cd206* gene expression (^##^*P* < 0.01 TM *vs*. TC and ^#^*P* < 0.05 TCM *vs*. TC), and the *Cd86/Cd206* mRNA ratio was not changed (Figure 3(d)). These results indicated that combinatorial cisplatin and moxibustion elevated the numbers of infiltrating CD8^+^ CTLs, CD4^+^ T, Th1, Th2, Th9 cells, Tregs, and M1 macrophages, as well as the expression of the *Cd69, Ifng, and Cd86* genes in the tumor microenvironment.

### 3.3. Combinatorial Cisplatin and Moxibustion Promoted Tumor Vascular Normalization

**Figure 4:**
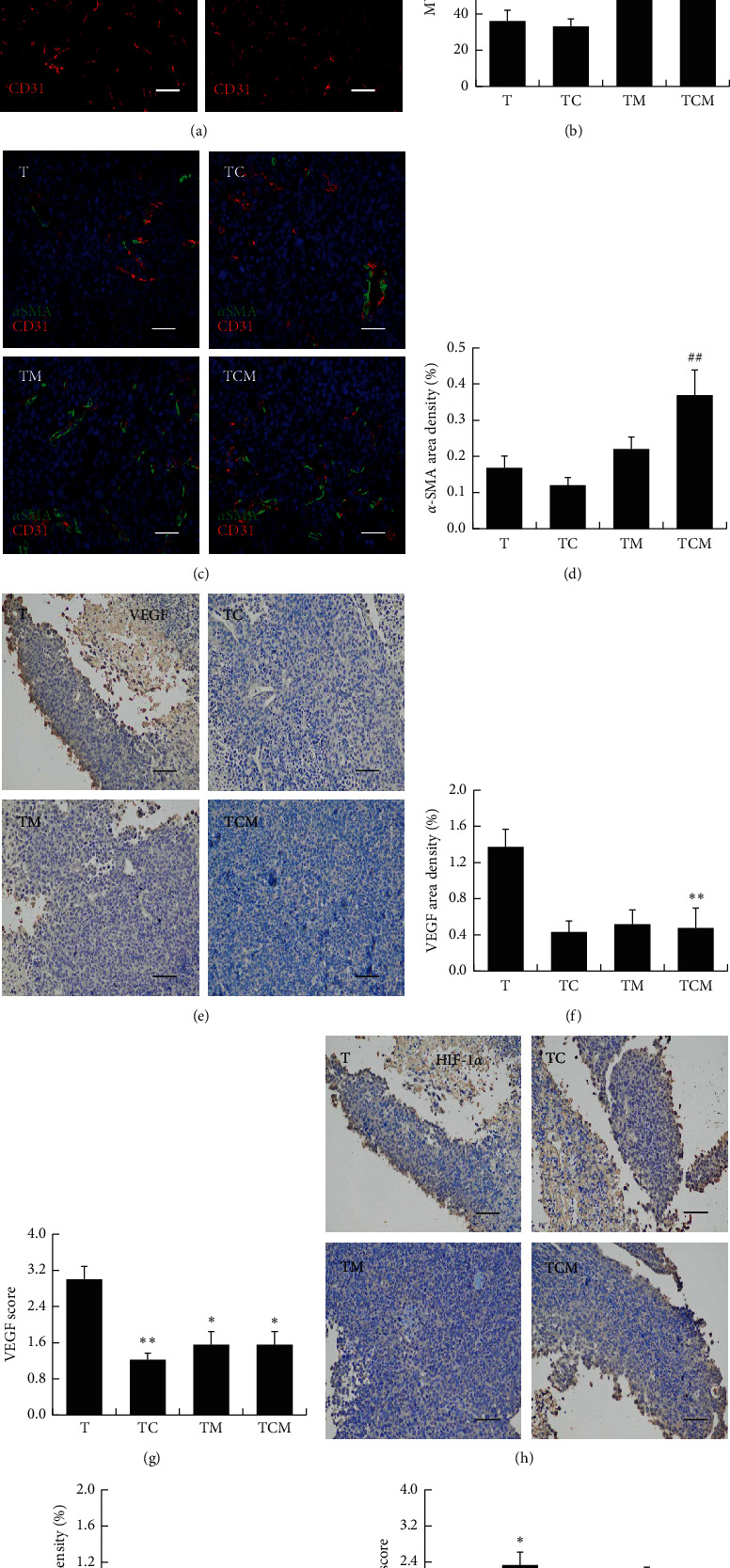
The effects of combinatorial cisplatin and moxibustion treatment on tumor vascular normalization. (a) Representative images of tumor microvessels showing CD31+ endothelial cells (red). (b) MVD values are shown by histograms representing quantitative image analysis of the immunofluorescence intensity in CD31+ endothelial cells (*n* = 3). (c) Representative images of the simultaneous immunodetection of endothelial cells (CD31, red), pericytes (SMA-*α*, green), and nuclei (DAPI, blue). (d) *α*-SMA area density showing pericyte coverage immunofluorescence intensity in *α*-SMA + pericytes (*n* = 3). Data in (b) and (d) are mean ± SEM, ^##^*P* < 0.01*vs*. TC. (e) Representative immunohistochemical staining for VEGF. (f-g) The semiquantification of VEGF expression is shown in (f) the area density of VEGF and (g) the VEGF score (*n* = 3). Data are mean ± SEM, ^*∗*^*P* < 0.05 and ^*∗∗*^*P* < 0.01*vs*. T. (h) Representative immunohistochemical staining for HIF-1*α*. (i-j) The semiquantification of HIF-1*α* expression is shown: (i) The area density of HIF-1*α*; (j) The HIF-1*α* score (*n* = 3). Data are mean ± SEM, ^*∗*^*P* < 0.05*vs*. T. Scale bars = 50 *μ*m in (a), (c), (e) and (h).

It is well known that tumor neovascularization is one of the main causes of tumor growth and metastasis [23]. As shown in Figure 4(a), CD31 was expressed on tumor-associated endothelial cells (TECs), and the LLC-bearing mice showed a high degree of vascular disorders, including distortion, dilation, and low blood vessel density. However, no significant improvement was seen after the cisplatin intervention while increased vessel density was found in the moxibustion and combinatorial therapy groups. The tumor MVD measurement reflects the number of blood vessels, as shown by the MVD measurements in CD31-stained sections (Figure 4(b)). The results showed that MVD was not changed by cisplatin but showed an increasing trend by moxibustion and combinatorial therapy. Decreased pericyte coverage is an additional feature of tumor vascular abnormality, and therefore, the pericytes surrounding TECs were investigated. As shown in Figure 4(c), there was a slight positive expression of *α*-SMA in tumor-bearing mice, which increased in mice treated with the combinatorial therapy. The semiquantitative analysis (Figure 4(d)**)** indicated that the *α*-SMA area density in the combined treatment group was higher than that in the chemotherapy group (^##^*P* < 0.01* vs*. TC). These results suggested that moxibustion intervention can improve the vascular structure of the tumor by increasing the pericyte coverage.

Abnormal neovascularization in the tumor microenvironment is due to the overexpression of proangiogenic factors, for instance, VEGF, which worsen the vascular normalization [24]. As shown in Figures 4(e)–4(g), the VEGF was strongly expressed in the tumor tissue of LLC-bearing mice but was weaker in the moxibustion, cisplatin, and combinatorial groups (Figure 4(e)). The semiquantitative analysis showed that, while the VEGF area density was significantly reduced with the combinatorial therapy compared with untreated mice (^*∗∗*^*P* < 0.01* vs*. T, Figure 4(f)), there was only a slight reduction in the cisplatin and moxibustion-treated mice. Further results showed that cisplatin, moxibustion, and combinatorial therapy could inhibit the VEGF score (^*∗∗*^*P* < 0.01 TC *vs*. T; ^*∗*^*P* < 0.05 TM and TCM *vs*. T, Figure 4(g)). Hypoxia induced by abnormal vascularization is associated with the growth of solid tumors. Therefore, we investigated the levels of the hypoxia-related marker HIF-1*α* (Figures 4(h)–4(j)**)**. However, no difference was found between the groups, except that the HIF-1*α* score was higher in cisplatin-treated tumors (^*∗*^*P* < 0.05* vs*. T, Figure 4(j)). These results indicated that combinatorial cisplatin and moxibustion treatment can improve vascular normalization in the tumor and suppress the expression of the tumor angiogenesis factor VEGF.

## 4. Discussion

In this study, analysis of the tumor growth curve, tumor weight, and tumor area showed that, while both cisplatin or moxibustion could effectively inhibit the tumor growth, the combinatorial therapy was significantly more effective (Figures 1(c)–1(f)). Cisplatin interferes with DNA lesions that inhibit tumor cell cycle progression and induce tumor cell death by apoptosis via both intra and interstrand crosslinking of DNA to bend and unwind the DNA duplex. A systematic evaluation has shown that moxibustion can reduce myelosuppression and the gastrointestinal toxicity of chemotherapy and radiotherapy, as well as improving organic immune function and the quality of life of cancer patients [11], but no clinical studies have reported direct inhibition of tumor growth by moxibustion. Consistent with our findings, it has been reported that moxibustion can improve the survival of tumor-bearing rodents. What is different is that those studies focused on the mechanism by targeting the inhibition of tumor cell proliferation and metastasis [25] and regulating the organic immune function [12, 26], while the current study concentrated on examination of the direct inhibition of tumor growth through the tumor microenvironment by moxibustion for the first time.

Recent findings have demonstrated the importance of the tumor immune microenvironment in the process of tumor development. Due to the immunosuppressive status of “cold” tumors (non-T cell-inflamed tumors), the enrichment of immunosuppressive cells promotes tumor progression. In contrast, tumor-infiltrating lymphocytes can recognize the immunogenic epitopes of tumor cells and activate the immune system, turning the tumor into a “hot” tumor (T cells-inflamed tumors) [27, 28]. High doses of chemotherapeutic drugs are known to cause immunosuppression with suppression of hematopoiesis in the bone marrow and thus depleting the sources of immune cells (including T cells), which may induce “cold” tumors and facilitate metastasis [5, 29]. Our study found that chemotherapeutic drugs could significantly reduce the net body weight of tumor-bearing mice, suggesting that the body condition deteriorated after cisplatin treatment (Figure 1(g)). Moreover, cisplatin could accelerate the infiltration of Treg immunosuppressive cells while not affecting CD8^+^ CTLs, CD4^+^ T cells (including the subsets Th1, Th2, Th9, and Th17 cells), and myeloid cells (subsets M1 macrophages, M2 macrophages, mDCs, and MDSCs, Figures 2(c) and 3(c)). These results are consistent with those of previous studies showing that chemotherapeutic drugs may worsen body function and aggravate the tumor immunosuppressive microenvironment [5, 29].

CD8^+^ CTLs are the most important effector cells for the recognition and clearance of tumor cells, but the tumor itself can incapacitate CD8^+^ CTLs by recruiting immunosuppressive cells such as TAMs and MDSCs [30]. CD4^+^ T cells are mainly divided into the immune-stimulating and immunosuppressive types. Among them, Th1 cells are the main cells that activate CD8^+^ CTLs by releasing IFN-*γ*, which promotes CD8^+^ CTLs recruitment to the tumor and the maintenance of CD8^+^ CTLs function [31]. Previous studies have reported that moxibustion increased the number of CD4^+^T and CD8^+^ T cells in the blood of patients with colon cancer metastasis to the liver, improving the immune status of patients [32]. Moxa-grain-moxibustion has been shown to promote the production of IL-2 which can promote the proliferation of lymphocytes in tumor-bearing mice [33]. In the present study, we focused first on the action of moxibustion on the tumor immune microenvironment finding that moxibustion and combinatorial therapy promoted the infiltration of Th1 cells as well as the gene expression of *Cd69* (a major marker of T cell activation) and the related cytokine *Ifng*. Furthermore, combinatorial therapy could promote the infiltration of CD8^+^ CTLs, CD4^+^ T cells, and Th9 cells (triggering CD8^+^ CTLs-mediated anti-tumor immunity through secretion of IL-9 and IL-21) in the tumor (Figures 2(c)–2(d)). These data further demonstrated that moxibustion may induce the “hot” tumor microenvironment. However, moxibustion could not restore the infiltration of Tregs induced by cisplatin.

Our results further showed that the combinatorial therapy promoted TAM polarization to the M1 phenotype and expression of the M1-type cell surface molecule *Cd86*, which is a positive costimulatory molecule that provides a signal for T cell activation and enhances the antitumor immune response [34]. However, the M1/M2 macrophages and *Cd86/Cd206* mRNA ratio were not changed by either cisplatin or moxibustion (Figures 3(c)–3(d)). M1 macrophages have been found to trigger the immune response by expressing a series of proinflammatory cytokines (including IL-23 and TNF-*α*) [35]. Furthermore, IFN-*γ* has been shown to convert anti-inflammatory TAMs into proinflammatory (M1-like) phenotypes that inhibit tumors [36]. Therefore, we speculate that combinatorial cisplatin and moxibustion may promote M1 macrophage polarization, followed by promoting CD8^+^ CTLs, Th1, and Th9 cell infiltration resulting in an anti-tumor immune effect mediated by the secretion of the tumor-killing cytokines.

An abnormal vascular system is, together with immunosuppression, one of the characteristics of the tumor microenvironment. Abnormal tumor vessels secrete growth factors and cytokines, as well as causing hypoxia, which promotes the tumor cells' survival and proliferation, and results in an immunosuppressive microenvironment [37]. The morphology of tumor angiogenesis is abnormal, visible as high levels of disorder, tortuousness, dilation, uneven thickness, excessive branching, low pericyte coverage, loose binding with TECs, and high interstitial fluid pressure. These characteristics result in vascular collapse and decreased blood flow, leading to the formation of an anoxic tumor microenvironment [38, 39]. Therefore, we further explored whether moxibustion and/or cisplatin could modulate tumor vascular abnormalities. The results showed that the tumor MVD and pericyte coverage shrank, and these abnormalities were not improved after cisplatin intervention. Both moxibustion and the combinatorial therapy could alleviate the tumor vascular abnormalities (Figures 4(a)–4(d)).

VEGF is overexpressed in different human tumors and has been verified as the most critical angiogenic factor in angiogenesis and vascular abnormalities leading to tumor progression [40]. It has been shown that low-dose VEGF and/or VEGF receptor antiangiogenic therapy can induce tumor vascular normalization, reduce hypoxia, promote tumor CD8^+^ T lymphocyte infiltration, and enhance tumor immunotherapy [41]. Our study further showed that moxibustion and combinatorial therapy could decrease the expression of the VEGF in tumor tissues (Figures 4(e)–4(g)), which may promote vascular normalization and CD8^+^ T cell infiltration to promote anti-tumor action.

The most direct cause of tumor neovascularization is the hypoxic state of the tumor. Activation of the HIF-1*α* transcription factor induces the generation of angiogenic factors to stimulate the growth of new blood vessels, providing nutrients for the tumor cells and allowing their survival in the hypoxic microenvironment [42, 43]. However, neither cisplatin nor moxibustion therapy could further upregulate the expression of HIF-1*α* (Figures 4(h)–4(j)), contrary to the regulatory effect of the VEGF and suggesting that improving hypoxia may not be a suitable therapeutic target for chemotherapy and moxibustion to inhibit angiogenesis.

In recent years, the interaction between tumor vascular remodeling and the reprogramming of the tumor immune microenvironment has been extensively studied. It has been shown that disruption of vessel normalization reduces the infiltration of T lymphocytes, depleting or inactivating CD4^+^ T lymphocytes as well as worsening the vascular normalization. Among them, Th1 cells that secrete IFN-*γ* may be the marker and decisive factor in immune checkpoint blocking and the antiangiogenic effect [44]. The M1 phenotype polarization in TAMs can normalize tumor blood vessels and enhance antitumor immunity as well [45, 46]. In view of these results, we speculate that combinatorial moxibustion and cisplatin treatment can enhance the immune-vascular crosstalk by increasing tumor infiltration of Th1 cells and M1 macrophages and the expression of Ifng mRNA, as well as promoting pericyte coverage leading to vascular normalization. However, the molecular mechanisms involved require further investigation.

## 5. Conclusion

To conclude, it was found that moxibustion could enhance the inhibitory effect of cisplatin on tumor growth by improving both the tumor immune microenvironment and vascular normalization. Combinatorial therapy could enhance the infiltration of CD8^+^ CTLs, CD4^+^ T cells, Th1, Th9 cells, and M1 macrophages in the tumor, as well as anti-tumor cytokine IFN-*γ*. In addition, combinatorial therapy improved vascular normalization, inhibited angiogenesis and VEGF expression and enhanced pericyte coverage. These results indicated that combinatorial cisplatin and moxibustion promote the mutual regulation of tumor vascular normalization and immune stimulation reprogramming, suggesting the potential of this combination for clinical application in improving the therapeutic efficacy of NSCLC treatment. Further investigation of the efficacy and mechanism of moxibustion combined with other therapies in different cancer types is planned.

## Figures and Tables

**Table 1:** The gene-specific primers used in this study.

Gene	Forward primer (5′⟶3′)	Reverse primer (5′⟶3′)	Product size
*Cd69*	AGTTTCTATCCCTTGGGCTGTG	AGCAACATGGTGGTCAGATGAT	136
*Il2ra*	AAAGCCCTCTCCTACAAGAACG	CTCGATTTGTCATGGGAGTTGC	149
*Foxp3*	CATCGTAGCCACCAGTACTCAG	TTGTGGAAGAACTCTGGGAAGG	141
*Ifng*	CATGGCTGTTTCTGGCTGTTAC	GTCACCATCCTTTTGCCAGTTC	145
*Tgfb1*	CCCCTATATTTGGAGCCTGGAC	GTAGTAGACGATGGGCAGTGG	130
*Cd86*	AAGTTGGTTCTGTACGAGCACT	ATACGAGCCCATGTCCTTGATC	141
*Cd206*	CAAACATTGGGCAGAAGGAGTG	CACATGCTGGTTTTACTGGTGG	102
*Actb*	CATCCGTAAAGACCTCTATGCCAAC	ATGGAGCCACCGATCCACA	171

## Data Availability

The data used to support the findings of this study are included within the article.

## Abbreviations

NSCLC: Non-small cell lung cancer
LLC: Lewis lung cancer
FBS: Fetal bovine serum
FCM: Flow cytometry assay
RT-qPCR: Real-time quantitative polymerase chain reaction
Tregs: Regulatory T cells
CD8^+^ CTLs: CD8^+^ cytotoxic T cells
BSA: Bovine serum albumin
VEGF: Vascular endothelial growth factor
HIF-1*α*: Hypoxia inducible factor-1*α*
CD31: Platelet and endothelial cell adhesion molecule-1
*α*-SMA: *α*-Smooth muscle actin
DAPI: 4′,6-Diamidino-2-phenylindole
IOD: Integrated optical density
MVD: Microvessels density
ANOVA: Analysis of variance
LSD: Least significant difference
IFN-*γ*: Interferon gamma
TAMs: Tumor-associated macrophages
mDCs: Myeloid dendritic cells
MDSCs: Myeloid-derived suppressor cells
TECs: Tumor vascular endothelial cells.
